# LSTM-Autoencoder for Vibration Anomaly Detection in Vertical Carousel Storage and Retrieval System (VCSRS)

**DOI:** 10.3390/s23021009

**Published:** 2023-01-15

**Authors:** Jae Seok Do, Akeem Bayo Kareem, Jang-Wook Hur

**Affiliations:** Department of Mechanical Engineering (Department of Aeronautics, Mechanical and Electronic Convergence Engineering), Kumoh National Institute of Technology, 61 Daehak-ro, Gumi-si 39177, Gyeonsang-buk-do, Republic of Korea

**Keywords:** anomaly detection, autoencoder, automatic storage and retrieval system, deep learning, long short-term memory, signal processing, vibration sensors

## Abstract

Industry 5.0, also known as the “smart factory”, is an evolution of manufacturing technology that utilizes advanced data analytics and machine learning techniques to optimize production processes. One key aspect of Industry 5.0 is using vibration data to monitor and detect anomalies in machinery and equipment. In the case of a vertical carousel storage and retrieval system (VCSRS), vibration data can be collected and analyzed to identify potential issues with the system’s operation. A correlation coefficient model was used to detect anomalies accurately in the vertical carousel system to ascertain the optimal sensor placement position. This model utilized the Fisher information matrix (FIM) and effective independence (EFI) methods to optimize the sensor placement for maximum accuracy and reliability. An LSTM-autoencoder (long short-term memory) model was used for training and testing further to enhance the accuracy of the anomaly detection process. This machine-learning technique allowed for detecting patterns and trends in the vibration data that may not have been evident using traditional methods. The combination of the correlation coefficient model and the LSTM-autoencoder resulted in an accuracy rate of 97.70% for detecting anomalies in the vertical carousel system.

## 1. Introduction

Industry 4.0, the fourth industrial revolution, has brought about significant changes in manufacturing and production processes by integrating cyber-physical systems and the Internet of Things (IoT). These technologies have enabled the creation of smart factories, where machines and systems can communicate and coordinate with each other to increase efficiency and productivity. However, the next phase of industrial development, known as industry 5.0, is expected to bring about even more significant changes by integrating artificial intelligence (AI) and machine learning. These will enable factories not only to coordinate and communicate with each other but also to adapt and learn from their experiences, leading to more flexible and efficient production processes [1,2,3,4,5]. One key aspect of industry 5.0 is using prognostics and health management (PHM) systems. These systems use data from sensors and machine learning algorithms to predict when equipment is likely to fail or require maintenance, allowing for proactive maintenance rather than reactive repairs. These not only help to increase equipment uptime but also reduce the risk of unplanned downtime, which can be costly for manufacturers. Another essential aspect of industry 5.0 is anomaly detection systems, which use data from sensors and machine learning algorithms to identify patterns that are out of the ordinary. These systems can help identify potential problems before they occur, allowing for early intervention and preventative measures. Integrating cyber-physical systems, AI, and machine learning in industry 5.0 is expected to bring about significant changes in manufacturing and production processes, leading to more efficient, flexible, and adaptable factories [6,7,8].

**Figure 1:**
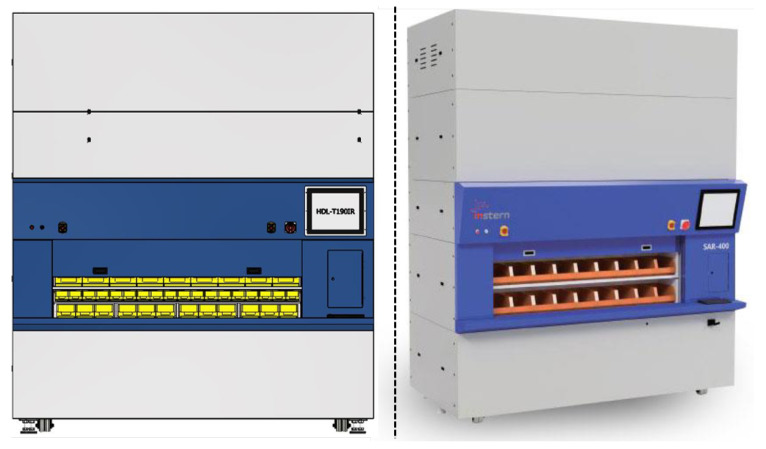
The front and side view of a vertical carousel module type of AS/RS system.

Many smaller businesses and retail warehouses formerly thought storage and retrieval systems were out of their price range since they lacked the funds to invest in such cutting-edge technology. However, since AS/RS technology has quickly developed over the years, new solutions offer a wide range of sizes, speeds, affordability, and flexibility, dramatically increasing the system adoption rate. As a result, AS/RS technologies are now among the most widely used and effective investment options available to most businesses [9]. The AS/RS system is made expressly to buffer, store, and retrieve merchandise and inventory as needed using the end user application. There are different types of the AS/RS system, namely, shuttles, cranes, carousels, vertical, lift modules, micro-loads, and unit loads. It can be easily integrated into any warehouse system and each industry deals with using a warehouse management system, warehouse execution system or different controls [10]. In this study, the point of focus is the vertical carousel module (VCM) type of AS/RS systems. Figure 1 depicts the front and side views of a typical vertical carousel storage and retrieval system (VCSRS). A vertical carousel module existed many years ago—a motor propels the carriers, which are made up of a number of them and are connected to a chain drive, in a vertical loop around a track, resembling a Ferris wheel.

Finding unusual occurrences, objects, or observations that are suspicious because they diverge dramatically from expected patterns or behaviors is known as anomaly detection. Other names for data anomalies include standard deviations, outliers, noise, novelty, and exceptions. It seeks to locate anomalous events—unexpected or infrequent events—in data streams. Detecting anomalies in the data can be beneficial both directly and as a starting point for knowledge discovery. Many applications, especially real-time ones where spotting anomalies is vital, such as those for security, critical infrastructure, and health, to name a few, depend on anomaly detection. Numerous strategies for outlier recognition, particularly unsupervised ones, are needed to identify this abrupt increase in activity as an outlier or rare object. A cluster analysis method, on the other hand, may frequently detect these microclusters more quickly. The anomaly detection techniques are unsupervised, semi-supervised, and supervised. The labels included in the dataset determine the appropriate anomaly detection method.

Combining the frequency spectrum and time domain analysis in real-world applications is highly desirable, particularly in rotating machinery. A complicated machine with numerous moving parts will produce a mixture of vibrations from the vibrations produced by all the moving parts. As a result, using simple time waveforms, it is challenging to assess the state of an extensive rotating equipment’s key components, such as gears, bearings, and shafts. Frequency analysis breaks down time waveforms and reveals how repeating vibration patterns are formed so that each component’s associated frequency can be explored. The fast Fourier transform (FFT) technique also permits efficient frequency analysis and the development of several different digital noise filters. The pre-definition of allowable operation vibration limits can be done by consulting current standards or long-term operation and maintenance data. The machine’s overall health may deteriorate, and defects may surface if the limit is exceeded. Frequency-domain vibration analysis excels at spotting unexpected vibrational patterns. For instance, a roller bearing with a cracked outer race will have frequent roller collisions. Usually, vibration from other sources obscures and covers up this information in time waveforms. By examining the frequency spectrum, it is possible to determine the regularity of the crashes and, consequently, the existence of bearing flaws. The contributions of the multi-sensor-based vibration anomaly detection study are highlighted below:

- A correlation coefficient approach of selecting the right features was adopted to ensure the selection of the right amount of sensors for the vibration study on the vertical carousel storage and retrieval system (VCSRS).
- Techniques such as filtering, normalization, and feature extraction are commonly used to improve the quality of the data and enhance the performance of the anomaly detection algorithms. These techniques have helped to remove noise and outliers, reduce dimensionality, and extract relevant features from the data, making it easier to detect patterns and anomalies. Overall, data preprocessing has played a crucial role in improving the accuracy and effectiveness of vibration anomaly detection for the vertical carousel module under study.
- The real-time multi-sensor vibration data from the vertical carousel storage and retrieval system have served as a prospect for the operational reliability of one of the AS/RS system module in a more efficient way.
- The PdM methodology assists with the current and future health monitoring status of the vertical carousel module under study, thereby predicting the anomalies as a result of misalignment from the brushless DC motor providing the rotational motion.

The structure of this paper is arranged as follows: Section 2 and Section 3 cover the literature review, related works, and theories of the proposed methodology. In contrast, Section 4 gives an overview of the proposed model for vibration-based anomaly detection, while Section 5 shows the sensor placement procedures and experimental setup for data collection. Section 6 discusses the frequency result, the threshold set for the anomalies, and the reconstruction error for the LSTM-autoencodermo del. Section 7 describes the conclusion and future work.

## 2. Literature Review and Related Works

Anomaly detection, also known as outlier detection, is crucial in various fields, such as cybersecurity, fraud detection, and fault diagnosis. Traditional anomaly detection methods, such as rule-based and statistical models, have limitations in handling complex and large-scale data. Recently, deep learning methods have been widely adopted for anomaly detection due to their ability to learn complex patterns and features from data. One popular deep-learning approach for anomaly detection is the use of autoencoders. Autoencoders are neural network models trained to reconstruct the input data by learning a compact representation. Anomaly detection using autoencoders assumes that normal data can be reconstructed with high accuracy while anomalous data cannot. The reconstruction error, calculated as the difference between the input and the reconstructed data, can be used as an indicator of the anomaly score.

Autoencoders have been applied to various anomaly detection tasks, such as intrusion detection in network traffic data [11] and fault diagnosis in mechanical systems [12,13]. Another deep learning method for anomaly detection is generative models, such as generative adversarial networks (GANs) and variational autoencoders (VAEs). These models can learn the distribution of normal data and generate new samples similar to the normal data. Anomaly detection using generative models is based on the assumption that anomalous data are unlikely to be generated by the model. The anomaly score can be calculated as the distance between the anomalous data and the generated samples. GANs and VAEs have been applied to anomaly detection tasks, such as fraud detection in financial transactions [14] and fault diagnosis in power systems [15].

In addition to autoencoders and generative models, other deep learning methods, such as convolutional neural networks (CNNs) and recurrent neural networks (RNNs), have also been used for anomaly detection. CNN’s have been applied to image-based anomaly detection tasks, such as identifying defects in manufacturing processes [16] and detecting abnormalities in medical images [17]. RNNs have been applied to time-series-based anomaly detection tasks, such as identifying abnormal behavior in network traffic data [18] and detecting anomalies in sensor data [19]. In summary, deep learning methods have shown promising performance in various anomaly detection tasks. However, there are still challenges to be addressed, such as the limited interpretability of deep learning models and the need for a large amount of labeled data for training. Further research is needed to improve the performance and robustness of deep learning-based anomaly detection methods.

Anomaly detection methods can be categorized into model-based and data-driven categories. Anomalies are typically divided into additive and multiplicative modes in the model-based fault detection method. It can be created by keeping an eye on how closely the measured outputs of the actual system match those of the model. The difference’s residuals can be used to represent it and then assessed to find flaws. The observer-based methods, parity-space methods, and parameter estimation strategies are examples of existing methodologies. A model that accurately depicts the system’s (expected) behavior is necessary for model-based anomaly identification. The model must include those system components necessary to address the current anomaly detection task. Some related works to model-based anomaly detection are [20,21,22]. Anomaly, novelty, and outlier identification in machine learning are frequently linked to data-driven fault detection. The method can be used in supervised, semi-supervised, and unsupervised settings. A training dataset is necessary for supervised anomaly identification by ML engineers. The dataset’s elements are divided into two groups: normal and faulty/abnormal. The model will use these examples to derive patterns from the previously unobserved data and enable it to recognize aberrant patterns. The caliber of the training dataset is crucial to supervised learning.

The most prevalent type of anomaly detection is neural networks (NN) and are the most well-known example of unsupervised algorithms. By eliminating the requirement for human labeling, artificial neural networks can reduce the manual labor required to preprocess instances. Even unstructured data can be processed using neural networks. NNs can spot irregularities in unlabeled data and apply what they have learned to new data. Methods for semi-supervised anomaly detection combine the advantages of the first two techniques. Engineers can use unsupervised learning techniques to work with unstructured data and automate feature learning. However, by combining AI with human oversight, they can watch and manage the trends the model picks up. This usually improves the model’s predictions [23,24,25,26,27,28,29]. In [30], they proposed the autoencoder and LSTM for traffic flow prediction. Their findings revealed high prediction accuracy using the regression metrics (RMSE, MRE, MAE). There has not been much research towards anomaly detection for major industrial systems such as automatic storage and retrieval systems. However, there are other related works that have adopted deep learning models compared to the traditional machine learning model for anomaly detection [31,32,33,34,35,36].

## 3. Theoretical Backgrounds

### 3.1. Modal Analysis

#### 3.1.1. Correlation Coefficient

Correlation coefficients measure the strength and direction of the relationship between two variables. The Pearson correlation coefficient, Spearman rank-order correlation coefficient, and Kendall rank correlation coefficient are three commonly used correlation measures. Interestingly, the Pearson correlation coefficient measures the linear relationship between two variables. It is defined as the covariance of the two variables divided by the product of their standard deviations. The Pearson correlation coefficient can range from −1 to 1, where −1 indicates a strong negative linear relationship, 0 indicates no linear relationship, and 1 indicates a strong positive linear relationship. It is typically displayed as ρ. However, the Spearman rank-order correlation coefficient is a nonparametric measure of the strength of the monotonic relationship between two variables. It is based on the ranks of the data rather than the raw data values themselves. Like the Pearson correlation coefficient, the Spearman correlation coefficient can range from −1 to 1, where −1 indicates a solid negative monotonic relationship, 0 indicates no monotonic relationship, and 1 indicates a strong positive monotonic relationship. Furthermore, the Kendall rank correlation coefficient is another nonparametric measure of the strength of the monotonic relationship between two variables. It is based on the number of concordant and discordant pairs in the data. The Kendall correlation coefficient can range from −1 to 1, where −1 indicates a solid negative monotonic relationship, 0 indicates no monotonic relationship, and 1 indicates a strong positive monotonic relationship.

Conclusively, the Pearson correlation coefficient is most appropriate when the relationship between the two variables is linear, while the Spearman and Kendall correlation coefficients are more appropriate for nonlinear relationships. The Pearson, Spearman, and Kendall mathematical expressions are represented by Equations (1)–(3), respectively. Several of these correlation coefficient techniques have been used in the literature to reduce computational costs, extract meaningful features for diagnostics and prognostics of various industrial systems, and also ensure that discriminative features are chosen for training and testing machine learning models [37,38,39,40,41].

(1) $$\rho_{p}=\frac{n(\sum xy)-(\sum x)(\sum y)}{\sqrt{\left[n\sum x^{2}-{\left(\sum x\right)}^{2}\right]\left[n\sum y^{2}-{\left(\sum y\right)}^{2}\right]}}\;$$

(2) $$r_{s}=1-\frac{6\sum d_{i}^{2}}{n(n^{2}-1)}\;$$

(3) $$t_{b}=\frac{P-Q}{\sqrt{\left(P+Q+X_{0}\right)}\left(P+Q+Y_{0}\right)}\;$$

#### 3.1.2. Fisher Information Matrix

The Fisher Information Matrix (FIM) is a powerful tool used in statistics and information theory to measure the amount of information contained in a probability distribution. It is commonly used in estimation theory to determine the best estimator for a given parameter and to calculate the lower bound of the variance of any unbiased estimator. The FIM is the expected value of the negative second derivative of the log-likelihood function concerning the distribution parameters. It is a symmetric and positive-definite matrix that can be used to calculate the Cramer–Rao lower bound, giving the minimum variance of any unbiased parameters estimator. The FIM is also used in hypothesis testing to determine the power of a test and in a decision theory to determine the optimal decision rule. Overall, the Fisher Information Matrix plays a vital role in understanding the information in a probability distribution and can be used to make informed decisions based on that information. It is the matrix of the score vector’s second cross-moments. It is a vector of the log-likelihood function’s initial partial derivatives with regard to its parameters [42,43]. The FIM equation of i-th participant can be expressed as follows in Equations (4) and (5):

(4) $$F_{i}\left(\xi \right)=E[(\frac{\partial }{\partial \xi }logL\left(\xi |y_{i}\right)\left(\frac{\partial }{\partial \xi }logL{\left(\xi |y_{i}\right)}^{t}\right]\;$$

FIM is affected by parameter values and stimulus (and, of course, the model). The stimulus symbol is typically omitted in the Fisher information matrix as depicted above; however, keeping in mind that the Fisher information is dependent on experimental design and stimuli. Otherwise, the equation given below is applicable if a true model is known:

(5) $$E\left[\left(\frac{\partial }{\partial \xi }logL\left(\xi |y_{i}\right)\right)\left(\frac{\partial }{\partial \xi }logL{\left(\xi |y_{i}\right)}^{t}\right)\right]=-E\left[\frac{\partial^{2}}{\partial \xi^{2}}logL\left(\xi |y_{i}\right)\right]\;$$

#### 3.1.3. Effective Independence

The theoretical analysis of the effective independence (EI) [44] approach begins with an efficient unbiased estimator of the modal coordinate *q*, and the estimate error covariance matrix can be written as follows in Equation (6):

(6) $$E\left[\left(q-\hat{q}\right){\left(q-\hat{q}\right)}^{T}\right]={\left[{\left(\frac{\partial y}{\partial q}\right)}^{T}{\left[\psi_{0}^{2}\right]}^{-1}\left(\frac{\partial y}{\partial q}\right)\right]}^{-1}\;$$

where *E* is the expected value and *q* is an efficient unbiased vector estimator; *q*, *y* are measurement column vectors indicating which structural positions are measured, and $\psi_{0}^{2}$ is the variance of the stationary Gaussian measurement white noise ε.

### 3.2. Vibration Signal Processing

#### 3.2.1. Fast Fourier Transform

The fast Fourier transform (FFT) is a widely used signal processing technique that efficiently computes the discrete Fourier transform (DFT). It has been extensively applied in various fields, such as vibration analysis (VA), anomaly detection(AD), and speech recognition (SR). Vibration analysis is the study of mechanical oscillations in structures and machines, which can provide valuable insights into the condition and performance of these systems. The FFT is a helpful tool in this field as it can decompose a vibration signal into its frequency components, which can be used to identify and analyze specific frequencies of interest. Ideally, the FFT can detect the presence of fault frequencies in a vibration signal, which can indicate an impending failure or malfunction in the system.

By combining the ability of the FFT to decompose a signal into its frequency components with the pattern recognition capabilities of deep learning, these methods can provide a powerful tool for identifying and analyzing mechanical issues in complex systems. Many vibration-related issues arise at specific frequencies, it is also possible to identify the source and location of the vibration based on variations in amplitude at those frequencies. By assuming that N (the length of the signal) is a multiple of 2, fast Fourier transforms (FFT) drastically minimize the number of intricate computations that must be performed. This assumption’s underlying mathematics eliminates unnecessary calculations and calculations with no value (such as multiplication by “1”), resulting in significant computational savings and reduces the number of operations to $N*log^{2}\left(N\right)$, which is a lot less than $N^{2}$. As a result, the fast Fourier transform can approximate discrete Fourier transforms that take more time to compute while doing so much more quickly [45,46,47]. The FFT algorithm can be expressed in Equation (7):

(7) $$X_{k}=\sum_{n=0}^{N-1}x_{n}\cdot e^{-i\frac{2\pi }{N}kn}$$

where $X_{k}$ is the *k*-th frequency component of the transformed sequence. $x_{n}$ is the *n*-th element of the original sequence. *N* is the length of the original sequence. *i* is the imaginary unit. The FFT algorithm works by recursively dividing the original sequence into smaller subsequences and combining the results using complex arithmetic. This allows the FFT to compute the DFT of a sequence in O(NlogN) time, which is much faster than the $O(N^{2})$ time required by the naive algorithm for computing the DFT.

Recently, deep learning methods have been applied in conjunction with the FFT for vibration analysis and anomaly detection. These methods, such as convolutional neural networks (CNNs) and recurrent neural networks (RNNs), can learn and recognize patterns in vibration signals that may not be immediately apparent to humans. Training these models on large datasets of normal and abnormal vibration signals can be used to accurately classify new vibration signals as normal or anomalous. One example of the use of deep learning in vibration analysis [48] utilized a CNN to classify vibration signals from a gearbox as either normal or anomalous. The authors found that their model achieved an accuracy of 97.6% in detecting gearbox anomalies, significantly outperforming traditional methods such as envelope spectrum analysis. FFT and deep learning methods have demonstrated outstanding vibration analysis and anomaly detection potential.

#### 3.2.2. Power Spectral Density

Engineers are inclined to analyze spectra using fast Fourier transforms (FFTs), but they should employ power spectral densities (PSDs). The rationale is that while PSDs are normalized to the frequency bin width, the length of the dataset (and associated frequency step) cannot affect the result’s amplitude. It is impossible for FFT to achieve this paradigm. In the real world, vibration is frequently “random” with a wide range of frequency components. To measure and contrast various vibration settings, power spectral densities (PSD, also known as acceleration spectral densities or ASD for vibration) are used. The relative intensity of vibration can be determined from time-domain data on vibration. However, the frequency or frequencies at which the energy is focused cannot be determined from time-domain data. The PSD, which depicts the power distribution of the time series data into frequency components, is what we need to obtain information in the frequency domain [49,50]. The discrete Fourier transform, *X*(*f*), of the VCSRS time-domain data, x(t), is used in the mathematical definition of the power spectral density function, or *X*_*PSD*_(*f*), which is defined in Equation (8) as:

(8) $$X_{PSD}\left(f\right)=\lim_{\Delta f\to \;0}\left[\frac{1}{2}\frac{X(f)X*(f)}{\Delta f}\right]$$

### 3.3. Autoencoder

The category of artificial neural networks includes autoencoders. The autoencoder learns input data representation. The reconstruction side is also known, along with the reduction side. The decoder attempts to recreate the input data using the previously learned latent space representation with the least loss on the reconstruction side. The encoder, which contains an input layer and hidden layer, a bottleneck, which stores learned/compressed data, and a decoder, which begins with the hidden layer and concludes with the output layer. These are the three parts that typically make up an autoencoder. An unlabeled dataset can be framed as a supervised learning problem to produce an output hatx that represents the original input hatx. This network can be trained by decreasing the reconstruction error (x,$\hat{x}$), which gauges the discrepancies between an initial information (input sequence) and the resulting reconstruction sequence [51,52,53].

### 3.4. Long Short-Term Memory (LSTM)

Long short term memory (LSTM) is a type of recurrent neural network (RNN) capable of learning long-term dependencies in data. Unlike traditional RNNs, which suffer from the vanishing gradient problem and cannot retain information over long periods, LSTMs can maintain a constant error flow through the network and preserve information over long periods.

The equation for the LSTM model can be defined using the mathematical expression below:

(9) $$i_{t}=\sigma \left(W_{i}*\left[h_{t-1},x_{t}\right]\right)+b_{i}$$

(10) $$f_{t}=\sigma \left(W_{i}*\left[h_{t-1},x_{t}\right]\right)+b_{f}$$

(11) $$C_{t}=tanh\left(W_{c}*\left[h_{t-1},x_{t}\right]\right)+b_{c}$$

(12) $$C_{t}=f_{t}*c_{t-1}+i_{t}*{\tilde{c}}_{t}$$

(13) $$\sigma_{t}=\sigma \left(W_{o}*\left[h_{t-1},x_{t}\right]\right)+b_{o}$$

(14) $$h_{t}=o_{t}*tanh\left(c_{t}\right)$$

where $x_{t}$ describes the input of the LSTM architecture cell, $h_{t-1}$, $h_{t}$, $c_{t-1}$ and $c_{t}$ represent the hidden states and cell states of the architecture which are documented in several related theories [54,55,56,57].

The key to LSTM’s ability to learn long-term dependencies is its use of three types of gates: input, output, and forget gates. These gates allow the LSTM to selectively remember or forget information and update the cell state based on the current input and previous state. The *input gate* determines which values will be updated in the cell state. It applies an activation function (sigmoid, ReLU, softmax) to the input and the previous hidden state, producing a vector of values between 0 and 1. These values are then multiplied element-wise with the input, which scales the input values according to their importance. The *forget gate* determines which values will be forgotten from the cell state. It applies a sigmoid function to the input and the previous hidden state, producing a vector of values between 0 and 1. These values are then multiplied elementwise with the previous cell state, which scales the previous cell state values according to their importance. The *output gate* determines which values will be output by the LSTM. It applies a sigmoid function to the input and the previous hidden state, producing a vector of values between 0 and 1. These values are then multiplied elementwise with the result of applying a hyperbolic tangent function to the current cell state, producing the output of the LSTM. In addition to the gates, LSTMs also have a cell state, a vector of values updated at each time step. The cell state is updated using the current input, the previous cell state, and the previous hidden state. The new cell state is then used to update the hidden state, which is used to make predictions. Overall, the LSTM architecture is well-suited for learning long-term dependencies in data. Its ability to selectively remember or forget information, and to update the cell state based on the current input and previous state, enables it to retain important information over long periods and make predictions based on this information [58,59,60].

## 4. Proposed Anomaly Detection Model

Anomaly detection is a technique for detecting odd patterns or instances in data that do not follow the expected pattern. It is frequently used in many industries, including cybersecurity, manufacturing, and healthcare, to detect odd events or observations that may suggest a problem or potential threat. Anomaly detection can be accomplished in a variety of ways, including through statistical methods, machine learning algorithms, and rule-based systems. Analyzing the statistical features of the data and spotting patterns that vary from the norm are all part of statistical approaches. In contrast, machine learning algorithms learn from data and detect patterns without prior knowledge of the underlying distribution. Rule-based systems, on the other hand, use predetermined rules to detect data anomalies. Some common techniques used in anomaly detection include [61,62,63]:

- One-class classification: This involves training a model on a dataset containing only normal instances and then using the model to identify instances that are significantly different from the normal instances.
- Outlier detection: This involves identifying instances that are significantly different from the majority of the instances in the dataset.
- Clustering: This involves grouping the data into clusters and then identifying instances that do not belong to any of the clusters.
- Time series analysis: This involves analyzing the data over time to identify unusual patterns or trends.

**Figure 2:**
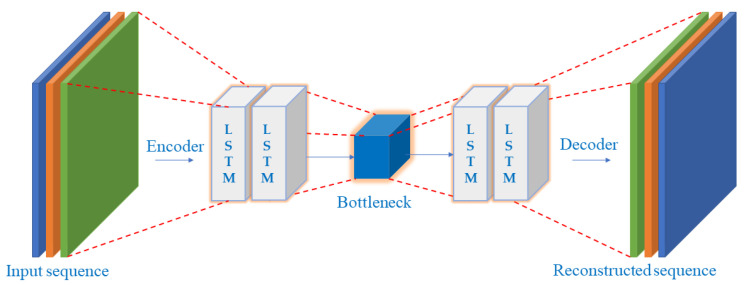
LSTM-Autoencoder Architecture.

**Figure 3:**
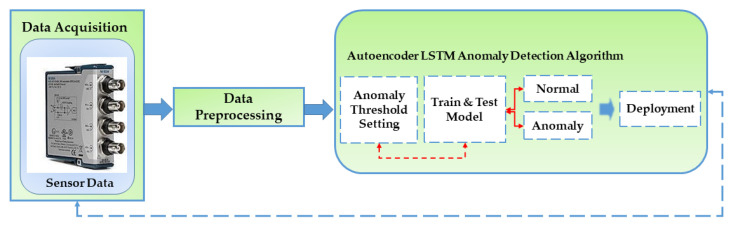
The LSTM-Autoencoder Model—a flowchart showing the steps from data acquisition to deployment.

The utilization of an autoencoder for sequence data utilizing an encoder-decoder LSTM architecture is known as LSTM-Autoencoder. The architecture is described in Figure 2. An encoder-decoder LSTM is set up to read the input sequence, decode it, and recreate it for a specific dataset of sequences. Based on the model’s capacity to replicate the input sequence, the model’s performance is assessed. The decoder component of the model may be eliminated, leaving only the encoder model after the model reaches the appropriate level of performance in replicating the sequence. Then, input sequences can be converted to a fixed-length vector using this paradigm. The generated vectors can then be applied to various tasks, including providing another supervised learning model with a compressed sequence representation. The autoencoder is responsible for gathering some input data, processing it via the model, and then producing a reconstruction of the input. The reconstruction ought to closely resemble the input. Utilizing minimal parameters will enable your model to learn a compressed version of the input. We will select a threshold at which a vertical carousel module is deemed abnormal and use that threshold to categorize a sequence as normal or anomalous. Losses from reconstructing the input as closely as feasible are the goal of training an autoencoder. This is accomplished by minimizing a loss function (just like in supervised learning). Reconstruction loss is the term for this function. Typical examples are mean squared error and cross-entropy loss [64,65]. The breakdown process and architecture for the proposed model are described in Figure 3. Table 1 describes the LSTM-Autoencoder hyperparameters used in this study.

## 5. Sensor Placement and Data Acquisition

Firstly, an overview of the modeling using a correlation coefficient technique to ascertain the placement of sensor is discussed. Two modeling techniques were deployed for better decision making: Effective Independence (Efi) and Fisher Information Matrix (FIM). Efi is a technique for removing sensors one at a time. At the same time, FIM is a technique for considering all possible scenarios up to the maximum determinant value determined when the number of sensors is fixed. It was discovered that the sensor positions applied with Efi differed from those applied with FIM, even though this approach uses FIM for repetitive computations. Only one node among the nodes with correlated mode forms is retained, and the remaining nodes are eliminated from the candidate group. The correlation coefficient compares the mode shapes of each node derived from the FEA result. Nodes with correlation coefficient values above the threshold set (0.7) were dismissed, leaving the Pearson, Spearman, and Kendall tau with 10, 11, and 25 sensors, respectively. Afterwards, an optimization was carried out following the lowest number of sensors extracted using the Pearson correlation [66].

### 5.1. Modeling Analysis Procedure

The overall modeling procedure is listed as follows:

- Use FEM to extract mode shapes of each node.
- Compute the correlation coefficient ρ for each node’s mode shapes.
- Set the threshold and once it is greater than ρ, one eliminates nodes. If it is lesser, one continues further the optimal sensor selection process to either effective independence (Efi) or Fisher information matrix (FIM).
- Lastly, perform a comparison of the EFi and FIM results.

**Figure 4:**
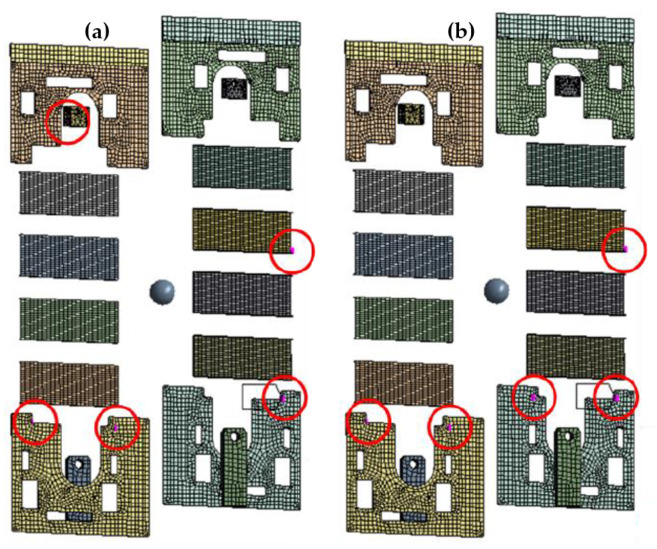
The optimal sensor placement result: (**a**) Pearson–Fisher Information Matrix, (**b**) Pearson Effective Independence.

Figure 4a shows the result of the optimal sensor placement for the Pearson-FIM result, identifying five different sensor position circles in red color. On the other hand, the Figure 4b shows the Pearson-Efi result for the optimal sensor placement, identifying five different sensor position for data collection. However, the researcher picked the modelling result for the Pearson-FIM because the area identified were evenly distributed compared to the Pearson-Efi. Both the location and the number of sensors are optimized by the correlation coefficient. Other algorithms analyze the mode forms of all nodes in a single repeated calculation, but the correlation coefficient considers only the mode shapes of two nodes in a single repeated calculation, resulting in significantly reduced computing performance. As a result, the sensor position could be optimized with less computational cost than traditional methodologies. The correlation coefficient has the disadvantage of being unable to optimize after determining the number of sensors; however, if the number of candidates excluded as a result of optimization is less than the intended number, only the threshold can be changed to determine whether the points should be excluded. As a result, even while recalculating, the computing requirements are minimal.

**Figure 5:**
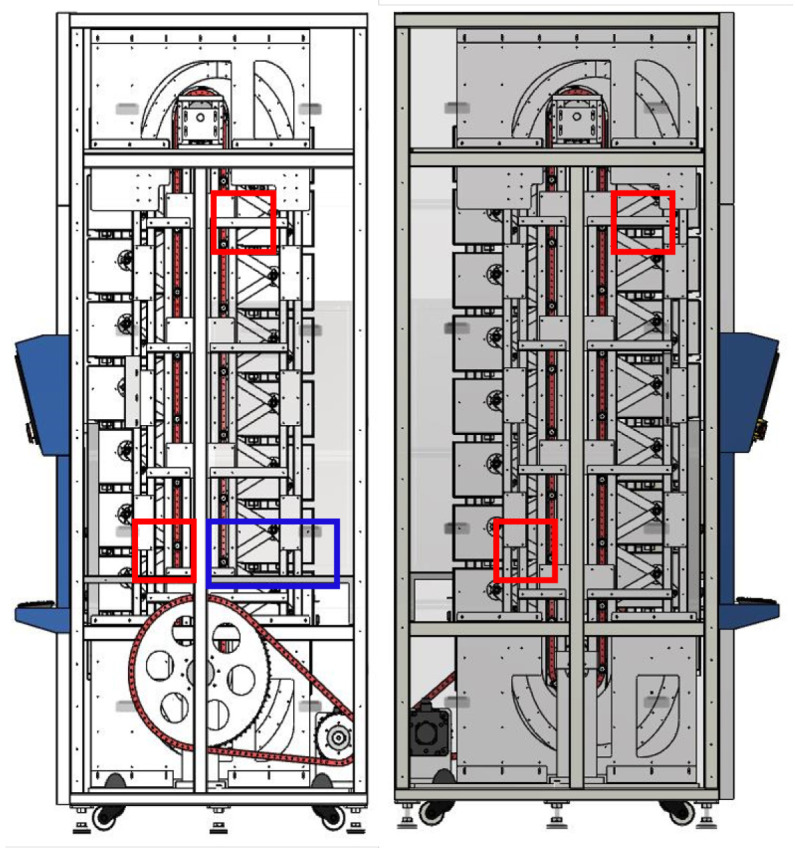
Identification of the vibration sensor placement on the vertical carousel storage and retrieval system.

**Figure 6:**
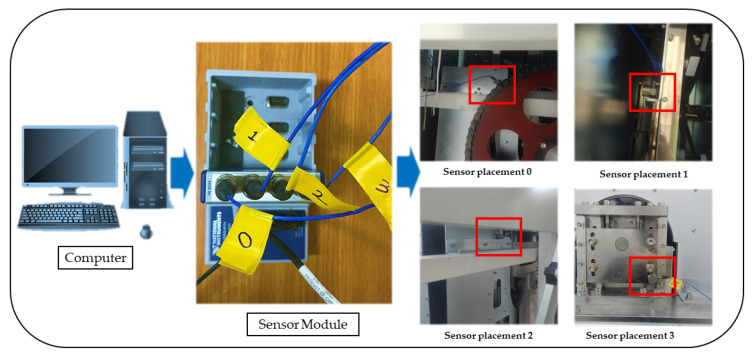
Pictorial view of the vibration sensor placement on the vertical carousel storage and retrieval system.

Furthermore, even if the number of sensors is known in advance, different methods can be employed in succession; the increased processing time in this scenario is insignificant. It is recommended to compare data acquired in various vibration settings once sensors are mounted at optimized spots [66]. Interestingly, Figure 5 and Figure 6 show the practical application and guide for the sensor placement. These have helped in the vibration-data acquisition process with the areas marked in blue and red boxes. With the SAR 400 being a ready-made product, the researchers were unable to place the vibration sensors as illustrated by the modelling result. Hence, the vibration sensor were placed close to the identified blue and red boxes. Looking closely, the vibration sensors were placed at the red box in the two figures (see Figure 5 and Figure 6) for better understanding while the blue box (see Figure 5 is left out of the vibration position based on the knowledge wise. Table 2 describes the dimension for the vertical carousel storage and retrieval system (VCSRS) produced by the Instern company, South Korea. Owing to the size of the product under study, the best pictorial view of the vibration sensors is shown in Figure 6, avoiding damages to the SAR 400 model.

### 5.2. Data Preprocessing

The data acquisition was carried out on the SAR-400 model vertical carousel storage and retrieval system (VCSRS) with vibration sensors placed as shown in Figure 5. The vibration data were collected at four vibration sensors using the NI 9234 model and processed using the python programming. The system specification includes 32 GB RAM, an NVIDIA GeForce RTX 3060 3.20 GHz processor, and a 64-bit operating system with an x64-based processor.

The most popular technique for assessing a vibration signal is frequency analysis. FFT helps transform a signal from the time domain into the frequency domain and is the most basic form of frequency analysis adopted by most researchers. A power spectrum, the result of this conversion, displays the energy present in particular frequencies of the total signal. This is quite helpful when examining stationary signals with constant frequency components. The findings of frequency analysis, such as a power spectrum or total harmonic distortion, only provide the frequency information of the signal, which has several drawbacks despite its widespread use. They do not have a time component. It indicates that signals whose frequencies change over time are unsuitable for frequency analysis. It is possible to take this concept further and say that an endless number of signals might all produce the same power spectrum. The frequencies of the top and bottom signals change over time, but in opposite directions. Although the frequency behavior of the two signals differs, the energy at each particular frequency in each signal is the same. Hence, their frequency spectra, as estimated by the FFT, are equal.

**Figure 7:**
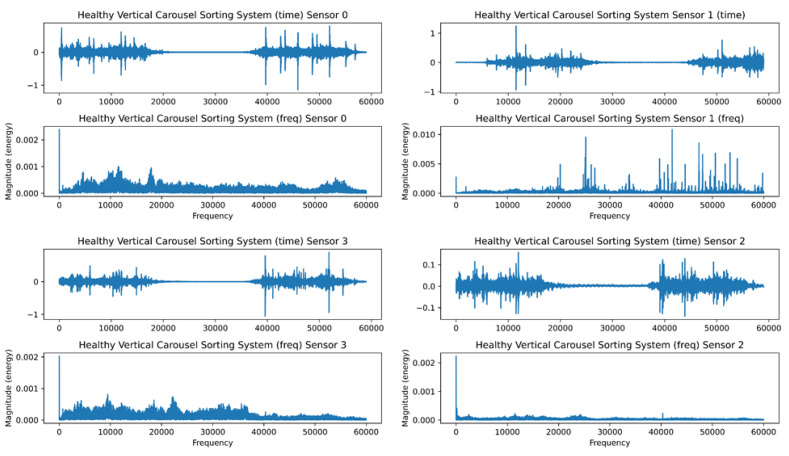
The healthy time and frequency analysis for the VCSRS vibration data.

**Figure 8:**
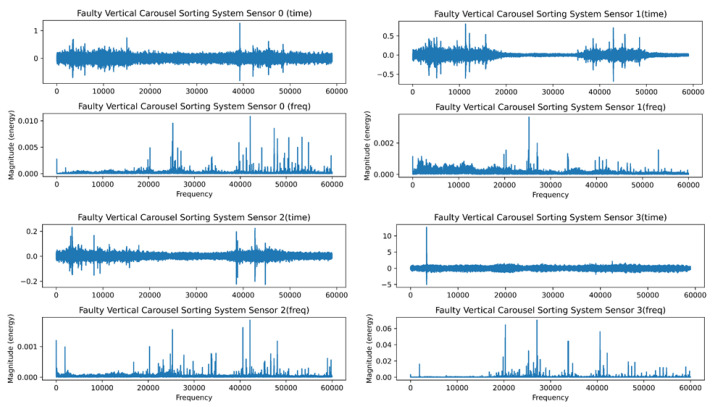
The faulty time and frequency analysis for the VCSRS vibration data.

The FFT’s inability to recognize transients or brief spikes in the signal is its second drawback. They typically have low energy and a broad frequency band. Transients’ energy is dispersed throughout a wide frequency range when converted into the frequency domain. Because of their low energy, it is impossible to detect transients in the frequency domain. Figure 7 and Figure 8 depict the healthy and faulty vibration data for the four installed sensors in the VCSRS, respectively. The sensors were labeled from 0 to 3, with 0 representing the first sensor, 1 representing the second sensor, 2 representing the third sensor, and 3 representing the fourth sensor. On each plot are the time-domain and the frequency-domain plots, with the same for the faulty vibration dataset. There were noticeable transients in sensor 1 compared with the other three sensors under the healthy vibration data. There were significant transients and spikes for all sensors in the faulty dataset.

**Figure 9:**
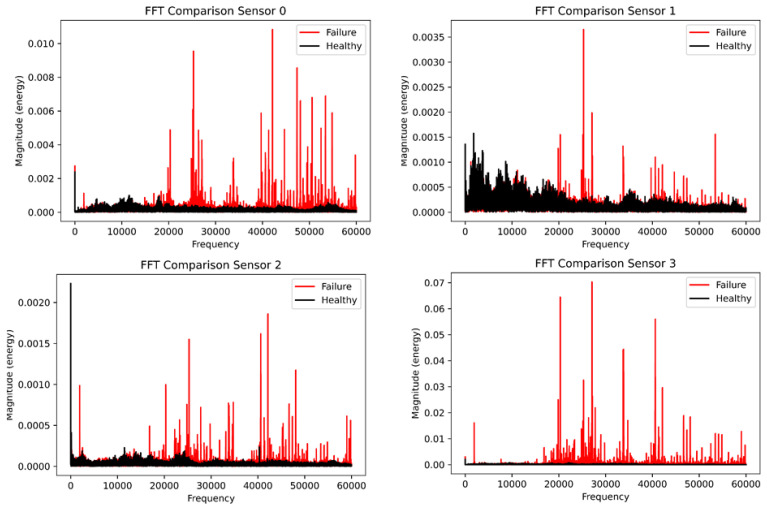
The healthy and faulty comparison using the fast Fourier Transform.

**Figure 10:**
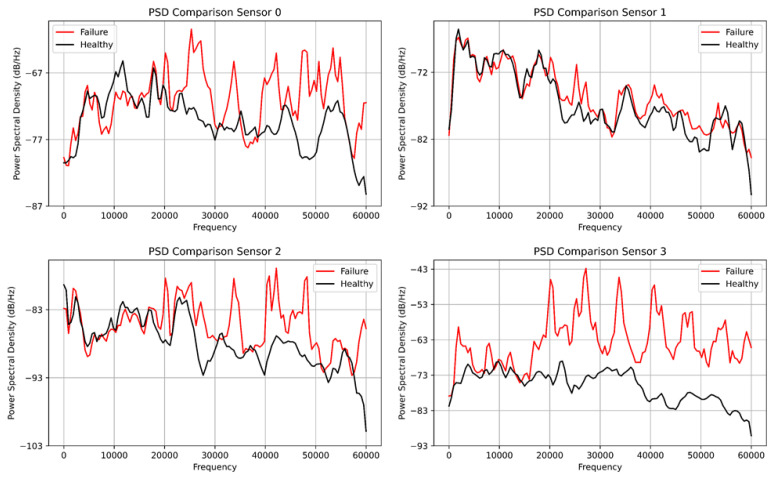
The power spectral density plot from the four sensor vibration datasets.

Figure 9 shows the comparison of the fast Fourier transform plot for both the healthy and faulty vibration datasets. Interestingly, the healthy dataset was colored in black and the faulty dataset was colored in red for all four plots. Sensor 0 shows significant transients for the faulty data compared with the healthy data, while sensor 1 had little transients for the healthy data but transients and spikes at 30,000 Hz compared with sensor 0 which had transient behavior at the tail end of the frequency plot. However, sensors 2 and 3 have evenly spaced spikes for the faulty data, while the healthy data have low energy for both sensors. Due to the setback of the FFT, we deployed the power spectral density to analyze the vibration data for the four sensors. Figure 10 displays the power spectral density plot with all sensors exhibiting significant behavior to distinguish the two datasets. With the FFT, we could not compare and quantify the vibration data, and PSD has been able to do justice to it.

### 5.3. Model Hyperparameter Function

Activation functions are used in the hidden layers of a neural network to introduce nonlinearity. Without nonlinear activation functions, a neural network would be just a linear regression model, which is not powerful enough to model most data. Some standard activation functions include sigmoid, tanh, and ReLU (Rectified Linear Unit). The sigmoid activation function maps any input to a value between 0 and 1, which is helpful for binary classification tasks. However, the sigmoid function can saturate for large positive or negative values, which can hinder learning. The tanh function is similar to the sigmoid function but maps input into values between −1 and 1. The ReLU function is becoming increasingly popular because it has been found to improve training speed and performance. The ReLU function maps all negative inputs to 0 and all positive inputs to their original value. This function is simple and computationally efficient, but it can suffer from the “dying ReLU” problem, where neurons stuck in the negative region do not contribute to the model’s predictions. Loss functions, also called cost functions, are used to measure the error between the predicted output of the model and the ground truth. Different loss functions are used for different types of tasks. For example, the mean squared error loss is often used for regression tasks, while the cross-entropy loss is used for classification tasks. The goal of training a deep learning model is to minimize the loss function by adjusting the weights and biases of the model. The mathematical expression for sigmoid, relu, and softmax are expressed in Equations (15)–(17), respectively [67,68].

(15) $$f_{x}=\frac{1}{1+e^{-x}}$$

(16) $$f_{x}=g_{x}=max\left(0,x\right)$$

(17) $$softmax\left(x_{i}\right)=\frac{exp(x_{i})}{\sum_{j}exp\left(x_{j}\right)}$$

### 5.4. Metrics for Model Performance

Several evaluation metrics are commonly used to assess the performance of a classification machine learning model. Some of the most popular ones are:

- Accuracy:
  This is the most intuitive and straightforward metric, which measures the fraction of correctly predicted instances over the total number of instances.
- Precision: It measures the fraction of positive instances that the model correctly predicts. It is useful when the cost of false positives is high.
- Recall: It measures the fraction of positive instances correctly predicted by the model out of all the positive instances in the data. It is useful when the cost of false negatives is high.
- F1 Score: It is the harmonic mean of precision and recall, and it is a balanced measure that considers both false positives and false negatives.
- AUC-ROC: It stands for the “Area Under the Receiver Operating Characteristic” curve and is a popular metric to evaluate the performance of a binary classifier. It represents the probability that a randomly selected positive instance will be ranked higher than a randomly selected negative instance.
- Confusion Matrix: A table that shows the number of true positives, true negatives, false positives, and false negatives in a classification problem. It is helpful to understand the types of errors that the model is making and to identify patterns in the data that the model cannot capture.
- Classification Report: It summarizes the evaluation metrics for a classification problem, including precision, recall, f1-score, and support (number of instances for each class).

It is essential to choose the appropriate evaluation metric depending on the characteristics of the problem statement. In some cases, it might be necessary to use a combination of different metrics to get a complete picture of the model’s performance [69,70]. The mathematical expression for accuracy, recall/sensitivity, precision, and recall are expressed in Equations (18)–(21).

(18) $$\;\mathrm{Accuracy}\;=\frac{TP}{TP+FP+TN+FN}$$

(19) $$\;\mathrm{Recall}/\mathrm{Sensitivity}\;=\frac{TP}{TP+FN}$$

(20) $$\;\mathrm{Precision}\;=\frac{TP}{TP+FP}$$

(21) $$\;F1-\mathrm{Score}\;=\frac{2*\mathrm{Sensitivity}*\mathrm{Precision}}{\mathrm{Precision}+\mathrm{sensitivity}}$$

## 6. Results and Discussion

An autoencoder is a neural network that learns a compact representation of input data called encoding. The autoencoder aims to reconstruct the input data when given the encoding. In other words, the autoencoder tries to learn a compressed representation of the input data and then generate output that is as close as possible to the input data. A long short-term memory (LSTM) is a type of recurrent neural network that can capture long-term dependencies in data. LSTMs are often used in natural language processing tasks, such as language translation and text generation. It is possible to use an LSTM as a part of an autoencoder architecture. In this case, the LSTM would be used to process the input data and learn a compact representation, which could then be used to reconstruct the original input data. The LSTM autoencoder would be trained using a reconstruction loss, which measures the difference between the original input data and the reconstructed output. The number of words in the input data does not necessarily impact the autoencoder’s performance. However, if the input data are extensive, it may be more difficult for the autoencoder to learn a good representation of it, as it will have more information to process. In general, an autoencoder’s performance will depend on various factors, including the size of the network, the quality of the training data, and the choice of hyperparameters.

LSTM-autoencoders can be trained using stochastic gradient descent and backpropagation, just like any other neural network. The network’s performance is typically evaluated using reconstruction loss, which measures the difference between the input and output sequences [71,72,73]. To validate the effectiveness of the proposed model for the anomaly detection of the normal and abnormal faults of the vertical carousel storage and retrieval system (VCSRS), the method is applied to the vibration signals collected from the position identified in Figure 5. The LSTM part of the architecture is used to capture long-term dependencies in the data, while the autoencoder part is used for dimensionality reduction and feature learning.

**Figure 11:**
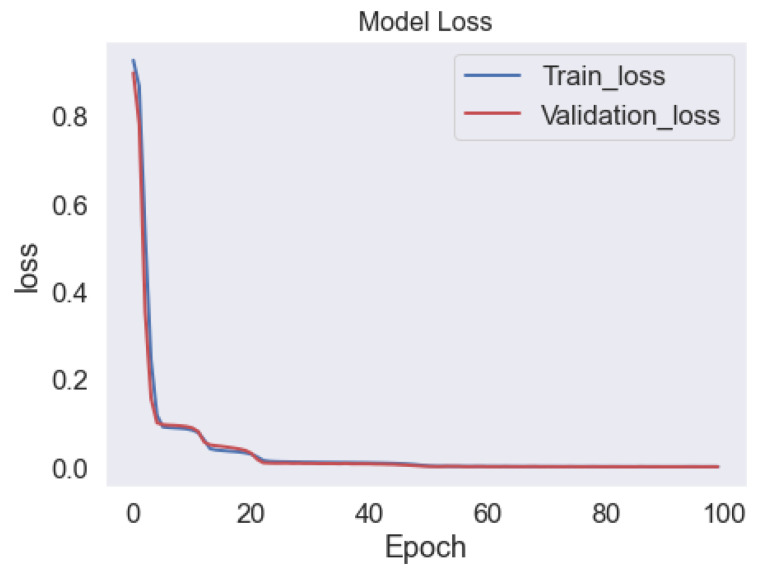
Training and validation Loss of the LSTM-autoencoder Model.

The loss function used in the training process of an LSTM-autoencoder is typically the mean squared error (MSE) between the input and the output of the network. The MSE measures the average squared difference between the predicted output and the true output. The goal of training is to minimize the MSE, which means that the network is trying to learn to reconstruct the input data as accurately as possible. During the training process, the model is usually trained on a set of labeled training data and its performance is evaluated on a separate set of validation data. The training loss is the loss calculated on the training set, while the validation loss is the loss calculated on the validation set. It is important to monitor the trend of these two losses during the training process, as a significant gap between the training loss and validation loss might indicate overfitting. A model that is overfitting has learned the training data too well, so it does not perform well on unseen data. There are multiple techniques to tackle overfitting, such as using L2 regularization, early stopping, or dropout. Figure 11 shows the training and validation loss for the LSTM-autoencoder model.

Additionally, it is common to use other metrics such as precision, recall, F1-score, or accuracy to evaluate the performance of the LSTM-autoencoder. However, those metrics would only be useful if one had the true label of the input data and was trying to classify the signal. The architecture of the LSTM-autoencoder consists of one input layer, two encoder LSTM, one repeated vector layer, two decoder LSTM, and one time distributed layer, resulting in seven layers and 63,492 parameters for the proposed model. The LSTM-autoencoder model is properly described in Table 1 showing the dropout rate at 0.1, epoch number (50), batch size (128), number of layers (8), number of classes (2), and number of units (50). Adaptive optimizers such as Adam are recommended to better handle the complicated training dynamics of recurrent neural networks (which a simple gradient descent may not address). The loss terms are added along the sequence and then divided by the maximum length of the sequence. The loss will be averaged out over the batch, making it easier to reuse the hyperparameters between tests [74,75,76,77].

**Figure 12:**
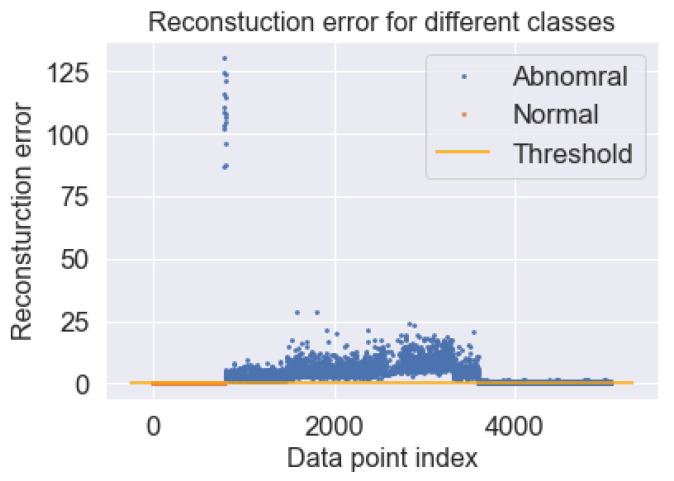
Visualization for the set threshold with reconstruction error for different classes.

**Figure 13:**
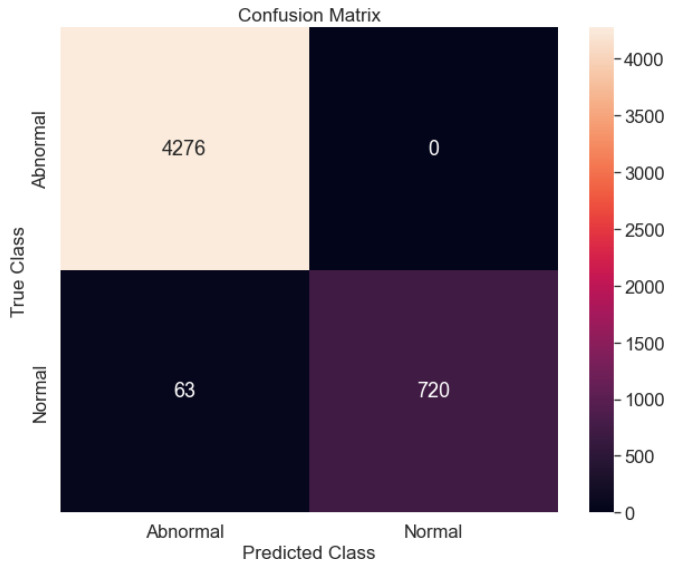
The confusion matrix for the anomaly detection proposed model between the normal and abnormal class.

The performance of the proposed model is checked using the thresholds set for normal and abnormal data. The set threshold is in yellow, while the normal and abnormal data are in blue and red, respectively. It can be seen that there is a big difference between the normal and abnormal classes, as shown in Figure 12. The y-axis labeled reconstruction error and the x-axis labeled data point depict the set threshold for the two classes. The confusion matrix plot, as shown in Figure 13 depicts the normal and abnormal classes with the true class on the y-axis and the predicted class on the x-axis. The proposed model was able to predict the abnormal class (4276) perfectly, while the proposed model predicted the normal class (730) and the abnormal class (53). The test accuracy figure is 97.70% which shows that the proposed model had a good prediction for anomalies. Table 3 shows the other evaluation metrics for the proposed model with a precision (100%), recall (95.20%), F1-score (92.43%) and computational cost (. However, future work will look into comparing the proposed model against other deep learning models. In addition, the problem of imbalanced data would be looked into, as it could be one of the reasons we had good accuracy for the proposed model. Overall, we have been able to propose a methodology that could detect anomalies using vibration data collected from four different sensors at different positions on the vertical carousel storage and retrieval system (VCSRS). Interestingly, this study serves as a continuation work to a study in [66].

The limitation of this study is the issue of data imbalance. There are a few different approaches addressing unbalanced datasets in machine learning. One approach is to oversample the minority class or undersample the majority class. This involves adding more copies of the minority class to the training set or removing some instances of the majority class. Another approach is to use a cost-sensitive learning algorithm, which means that the algorithm takes into account the costs of misclassifying different classes. This can be done by assigning different weights to different classes or by using a different cost function during training. Another option is to use a different evaluation metric, such as precision, recall, or the F1 score, which are more sensitive to imbalanced class distributions than accuracy. Finally, one can also try using a different machine learning algorithm that is less sensitive to imbalanced data, such as a decision tree or a random forest [78,79].

## 7. Conclusions

In conclusion, this study successfully demonstrated an autoencoder and long short-term memory model for anomaly detection using vibration data from four sensors in a vertical carousel storage and retrieval system (VCSRS). The primary driver of the VCSRS, the electric motor, was identified as the primary faulty component. The sensor placement process was carried out using the correlation coefficient techniques of the Fisher information matrix and effective independence, resulting in the placement of sensors in 4–5 specific areas. The methodology is designed to monitor the system with a focus on the electric motor through the vibration data collected from the four sensors attached to the vertical carousel module. The vertical carousel module motor propels the carriers in a vertical loop around a track in both forward and backward motions, resembling a Ferris wheel. The LSTM-autoencoder model has the following stages—first is the “model learning stage”, where the model’s training occurs using the data under normal working conditions of the VCSRS. The second phase is termed the “anomaly stage”, where the threshold of the LSTM-autoencoder anomaly detection is set. The accuracy of the LSTM-autoencoder model was found to be 97.70% due to tuning of various hyperparameters. Future work on this study includes comparing the proposed model with other deep learning models and addressing the issue of data imbalance. Additionally, it would be helpful to explore the potential for implementing the proposed model in a real-time monitoring system for the VCSRS, as this could significantly improve maintenance and operational efficiency. Overall, this study contributes to anomaly detection and serves as a valuable resource for those interested in using vibration data to predict failures in VCSRS and similar systems. The successful implementation of the autoencoder and long short-term memory model suggests that it could be a promising approach for other applications, as it gives room for different methodologies with the other component associated with the VCSRS (from further feedback from end users and industries) as this would further increase its working lifespan and avoid unscheduled shutdowns.

## Figures and Tables

**Table 1:** LSTM-Autoencoder Architecture Model Parameters.

Model Architecture	Description
Number of Classes	2
Number of Layers	7
Batch Size	128
Number of Epochs	100
Dropout Rate	0.001
Optimizer	Adam
Activation Function	Softmax, ReLU, and Sigmoid
Loss Function	MSE

**Table 2:** VCSRS Design Specification.

System	Item	Description
VCSRS Overall Spec.	Size	2820 W × 1540 L × 3280 H
	Full Load	2000 kg
	Speed	0.6 m/s
	Size	1900 W × 300 L × 200 H
Pallet Spec.	Quantity	20 EA
	Load Capacity	100 kg

**Table 3:** Evaluation metrics values for the LSTM-Autoencoder model.

Algorithm	Accuracy (%)	Precision (%)	Recall (%)	F1-Score (%)	Cost (s)
AE-LSTM	97.70	100.00	95.20	92.43	2517.8

## Data Availability

The data presented in this study are available on request from the corresponding author. The data are not publicly available due to laboratory regulations.

## Abbreviations

The following abbreviations are used in this manuscript:

AD	Anomaly Detection
AE	Autoencoder
AI	Artificial Intelligence
AS/RS	Automatic Storage/Retrieval System
CNN	Convolutional Neural Networks
DFT	Discrete Fourier Transform
EFI	Effective Independence
FFT	Fast Fourier Transform
FIM	Fisher Information Matrix
FN	False Negative
FP	False Positive
IoT	Internet of Things
LSTM	Long short term memory
MSE	Mean Square Error
NN	Neural Network
PHM	Prognostics and Health Management
PSD	Power Spectral Density
RELU	Rectified Linear Unit
RNN	recurrent Neural Networks
RUL	Remaining Useful Life
TN	True Negative
TP	True Positive
VA	Vibration Analysis
VCSRS	Vertical Carousel Storage and Retrieval System
